# A novel function for Egr4 in posterior hindbrain development

**DOI:** 10.1038/srep07750

**Published:** 2015-01-13

**Authors:** Chang-Joon Bae, Juhee Jeong, Jean-Pierre Saint-Jeannet

**Affiliations:** 1 Department of Basic Science & Craniofacial Biology, College of Dentistry, New York University, New York, USA; 2 Permanent address: Ministry of Food and Drug Safety, 187 Osongsaengmyeong2(i)-ro, Osong-eup, Cheongwon-gun, Chungcheongbuk-do 363-700, Republic of Korea

## Abstract

Segmentation of the vertebrate hindbrain is an evolutionarily conserved process. Here, we identify the transcription factor early growth response 4 (egr4) as a novel regulator of posterior hindbrain development in Xenopus. *egr4* is specifically and transiently expressed in rhombomeres 5 and 6 (r5/r6), and Egr4 knockdown causes a loss of *mafb/kreisler* and *krox20/egr2* expression in r5/r6 and r5, respectively. This phenotype can be fully rescued by injection of frog or mouse *Egr4* mRNA. Moreover Egr4-depleted embryos exhibit a specific loss of the neural crest stream adjacent to r5, and have inner ear defects. While the homeodomain protein vHnf1/Hnf1b directly activates *Mafb and Krox20* expression in the mouse hindbrain to specify r5, we show that in Xenopus this process is indirect through the activation of Egr4. We provide evidence that rearrangements in the regulatory sequences around *egr4* and *mafb* genes may account for this difference.

Development of the vertebrate hindbrain along the anteroposterior axis is an evolutionarily conserved process. It involves the transient formation of seven to eight transversal swellings known as rhombomeres (r). These territories are cell lineage restricted compartments and constitute developmental units for cranial motor nerves and neural crest cells. In most vertebrates, cranial neural crest cells originate from r2, r4 and r6 and migrate as three streams that populate the 1st, 2nd and 3rd branchial arches, respectively. Neural crest cells give rise to the ganglia of cranial nerves V, VII/VIII and IX, and to craniofacial skeletal elements unique for each branchial arch. Fewer neural crest cells are produced from r3 and r5; these cells typically migrate rostrally and caudally to join the adjacent streams^1^,^2^,^3^. This conserved pattern of neural crest cell migration directly depends on the proper segmentation of the hindbrain. Caudal hindbrain patterning is also critical for the specification and development of the inner ear which arises from the ectoderm adjacent to r4/r5^4^.

The gene regulatory network underlying hindbrain segmentation involves a complex interplay of transcription factors and signaling molecules leading to the regional expression of different combination of genes, which imparts a unique molecular identity to each rhombomere^5^. Several of these genes encode transcription factors, such as Krox20/Egr2, MafB/Kreisler/Valentino, Hnf1b/vHnf1 and members of the Hox family. In the posterior hindbrain, the zinc finger transcription factor Krox20 is expressed in the prospective r3 and r5^6^,^7^. The basic leucine zipper Mafb is expressed in r5 and r6, and controls the specification of these two rhombomeres, and activates Krox20 in r5^8^,^9^. The homeodomain transcription factor vHNF1 (variant hepatocyte nuclear factor 1) cooperates with FGF to promote caudal hindbrain identity, by activating Mafb and Krox20 expression in r5/r6 and r5, respectively^10^,^11^. Hoxb1 is expressed in the neural plate with an anterior limit at the prospective r3/r4 boundary, and later is only maintained in r4, where it acts with other Hox factors to specify r4 identity^12^. The regulatory cascade underlying rhombomere specification is highly conserved among vertebrates, and only minor variations in this blueprint have been reported.

Here we describe the function of the transcription factor early growth response 4 (Egr4), a novel regulator of hindbrain development in *Xenopus.* Egr4 is transiently expressed in r5/r6 at neurula stages. Using loss- and gain-of-function approaches we demonstrate that Egr4 is required downstream of Hnf1b to activate *mafb* and *krox20* expression and promote r5 identity in the posterior hindbrain. This is the first report describing a role for Egr4 in the vertebrate nervous system.

## Results

### *Xenopus laevis egr4* is a target of Pax3 and Zic1

**Figure 1:**
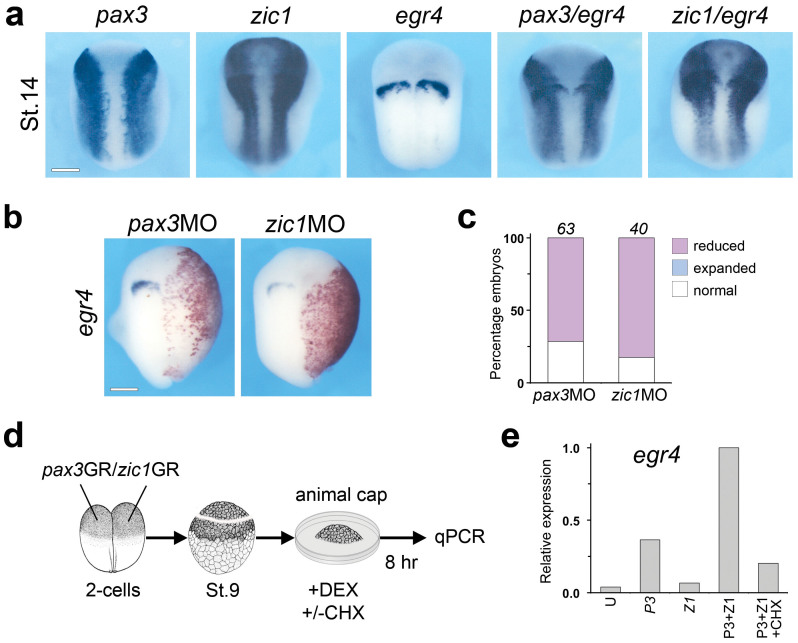
*egr4* is a downstream target of Pax3 and Zic1. (a) Whole-mount *in situ* hybridization for *egr4*, *pax3* and *zic1*. The DIG-labeled probes were used alone or in combination (*pax3/egr4 and zic1/egr4*). Dorsal views, anterior to top. Scale bar, 300 μm. (b) Injection in one blastomere at the 2-cell stage of MOs to block Pax3 (*pax3*MO; 40 ng) or Zic1 (*zic1*MO; 40 ng) function resulted in a reduction of *egr4* expression. The injected side is to the right as indicated by the presence of the lineage tracer (Red-Gal). Dorsal views, anterior to top. Scale bar, 300 μm. (c) The graph indicates the percentage of embryos with normal (white) or reduced/lost (red) *egr4* expression. The number of embryos analyzed is indicated on top of each bar. (d) mRNA encoding *pax3*GR and *zic1*GR (100 pg each), alone or in combination were injected into both blastomeres in the animal pole at the 2-cell stage. At the blastula stage (stage 9), animal cap explants were dissected and cultured for 8 hours in the presence of dexamethasone (DEX). In some samples, cyclohexamide (CHX) was used to block protein synthesis. (e) *egr4* expression in *pax3*GR and *zic1*GR injected animal cap explants analyzed by qPCR.

Egr4, a member of the early growth response (EGR) family of zinc-finger transcription factors, was identified in a microarray screen for targets of Pax3 and Zic1^13^, two transcription factors that are necessary and sufficient to specify the neural plate border in *Xenopus*^14^,^15^,^16^. *Xenopus laevis egr4* possesses an open reading frame encoding 486 amino acids. At the amino acid level, *Xenopus laevis* Egr4 shares 40% identity with human EGR4 (accession # NP_0011956), 44% identity with mouse Egr4 (accession # NP_065621), 50% identity with chicken Egr4 (accession # SP_420888) and 89% identity with *Xenopus tropicalis* Egr4 (accession # XP_004911346). By whole mount *in situ* hybridization *egr4* is detected in a single stripe across the neural plate, extending into the neural plate border where *egr4* overlaps with *pax3* and *zic1* expression domains (Fig 1a). To confirm that *egr4* is a target of Pax3 and Zic1, we performed perturbation experiments in the embryo using *pax3-* and *zic1*-specific morpholino antisense oligonucleotides (MOs)^14^,^15^,^16^. Pax3 or Zic1 knockdown resulted in a reduction or loss of *egr4* expression in most embryos (Fig 1b–c), suggesting that both factors are independently required for *egr4* expression. In animal cap explants from embryos injected at the 2-cell stage (Fig 1d), expression of *pax3*GR and/or *zic1*GR (the hormone-inducible versions of *pax3* and *zic1*, fused to the human glucocorticoid receptor ligand-binding domain) showed that Pax3 and Zic1 are sufficient to synergistically activate *egr4* (Fig 1e). Moreover, *egr4* expression in *pax3*/*zic1*-injected explants was dramatically reduced when the protein synthesis inhibitor cyclohexamide (CHX) was added to the culture medium, indicating that the induction of *egr4* by Pax3 and Zic1 is indirect (Fig 1e).

### Egr4 is specifically and transiently expressed in the posterior hindbrain

**Figure 2:**
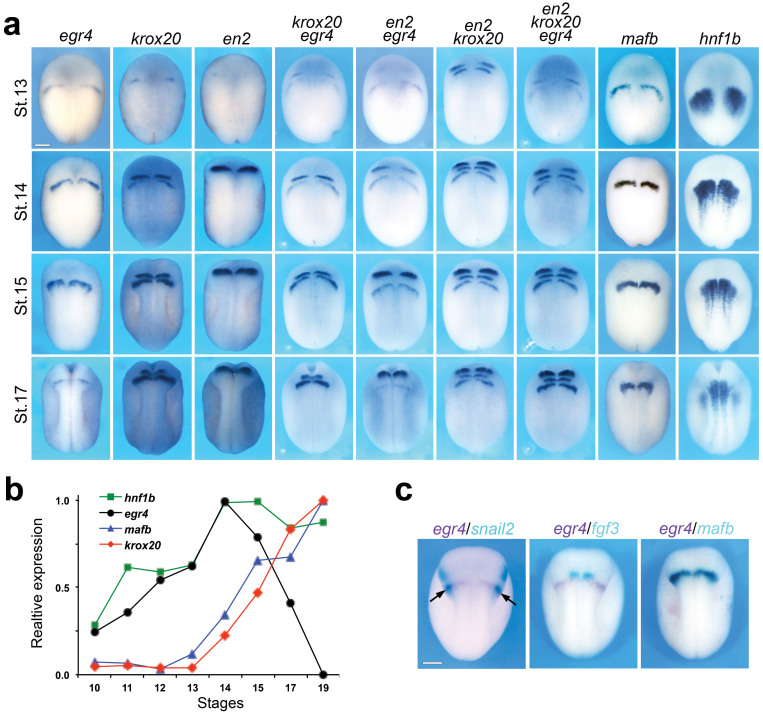
*egr4* is specifically and transiently expressed in r5 and r6. (a) Analysis of the spatiotemporal expression of *egr4* as compared to genes expressed in similar region of the ectoderm, including *en2* (midbrain-hindbrain boundary), *krox20/egr2* (hindbrain r3/r5), *mafb/kreisler* (hindbrain r5/r6) and *hnf1b/vHnf1b* (hindbrain r5/r6/r7). The probes were used alone or in combination as indicated. Scale bar, 300 μm. (b) Temporal dynamics of the expression of *egr4*, *krox20*, *mafb*, and *hnf1b* analyzed by qPCR in whole embryos at the indicated stages. The values were normalized to *odc1*. (c) Two-color in situ hybridization for *egr4/snail2*, *egr4*/fgf3, and *egr4*/*mafb* at the neurula stage. The expression of *egr4* at the neural plate border overlaps with the neural crest marker, *snail2* (arrows). *egr4* is detected immediately adjacent and posterior to *fgf3* expression domain in r4. *egr4* and *mafb* have overlapping expression domains in r5/r6. Scale bar, 300 μm.

In order to more precisely map the expression domain of *egr4*, we performed *in situ* hybridization on embryos at different stages using digoxigenin-labeled RNA probes for a number of genes expressed in similar region of the ectoderm, including *en2* (midbrain-hindbrain boundary^17^), *krox20/egr2* (hindbrain r3/r5^18^), *fgf3* (hindbrain r4^19^) *mafb/kreisler* (hindbrain r5/r6^20^), *hnf1b/vHnf1b* (hindbrain r5/r6/r7^21^) and *snail2* (neural crest^22^). The probes were used alone or in combination as indicated (Fig 2a). *egr4* expression is initiated at stage 13 as a single domain across the neural pate. This expression progressively increases to reach a maximum around stage 14–15, and then slowly decreases to become undetectable around stage 19/20 (Fig 2a; not shown). This expression profile was independently confirmed by qPCR (Fig 2b). Spatially, there is no overlap between *egr4 and en2* expression domain, however, *egr4* is co-expressed with *krox20* in r5 (Fig 2a). Temporally, the escalation of *egr4* expression precedes *krox20* and *mafb* expression as shown by qPCR (Fig 2b). We also performed two-color *in situ* hybridization using *snail2*, *fgf3* and *mafb* in combination with *egr4* (Fig 2c). The most lateral domain of *egr4* overlaps with *snail2* demonstrating that *egr4* is expressed in a subpopulation of neural crest progenitors. In addition, *egr4* expression domain in the hindbrain lays immediately posterior to *fgf3* expression domain in r4, and overlaps completely with *mafb* in r5/r6 (Fig 2c). Taken together, these results indicate that *egr4* is specifically and transiently expressed in r5/r6, and its expression escalates ahead of the expression of *krox20* and *mafb* expression in this region.

### Egr4 is required for *mafb* and *krox20* expression in the posterior hindbrain

**Figure 3:**
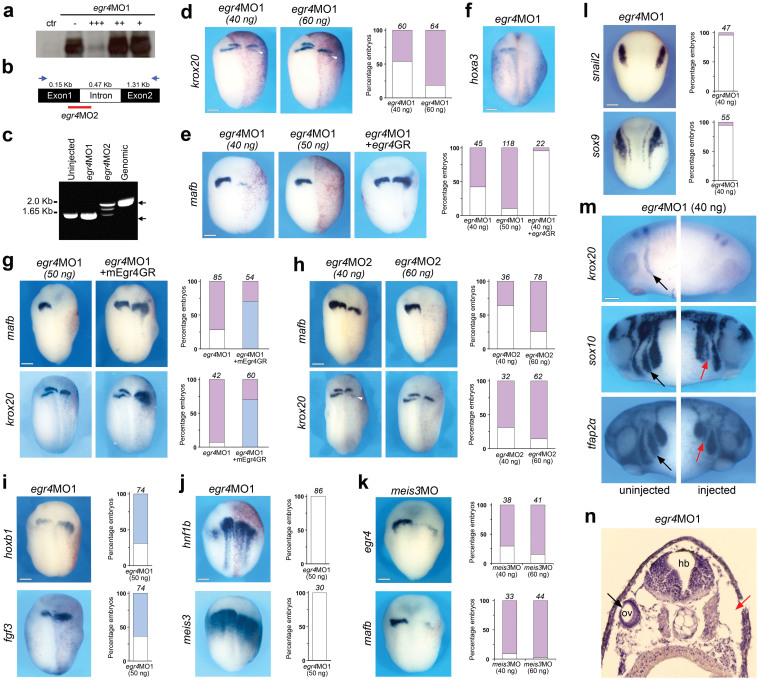
Egr4 is required for caudal hindbrain development. (a) Increasing amounts of *egr4*MO1 10 ng (+), 100 ng (++), and 1000 ng (+++) blocks translation directed by *egr4* mRNA in an *in vitro* coupled transcription/translation reaction. Full-length blot is presented in Supplementary Figure 1. (b) Schematic representation of the *egr4* locus. The PCR primers used for the analysis of spliced transcripts are indicated (blue arrows). The position of the splice (*egr4*MO2) blocking MO is indicated. (c) In *egr4*MO2-injected embryos a larger *egr4* transcript is detected (~2.0 kb) due to intron retention. For all samples, the RT-PCR was performed under the same experimental conditions. (d–f) Unilateral injection of *egr4*MO1 at the 2-cell stage caused a reduction of expression of *krox20* in r5 (d), *mafb* in r5/r6 (e) and *hoxa3* (f). Note the residual *krox20* in r5 (arrowheads). Injection of *Xenopus* egr4GR (e) or mouse Egr4GR (g) mRNAs (100 pg) can efficiently rescue *mafb* or *krox20* expression in *egr4*-depleted embryos. (h) Unilateral injection of *egr4*MO2 results in a specific reduction/loss of expression of *krox20* in r5 and *mafb* in r5/r6. Note the residual *krox20* in r5 (arrowhead; left panel). (i–j) Unilateral injection of *egr4*MO1 (50 ng) at the 2-cell stage caused a posterior expansion of *hoxb1* and *fgf3* (i), while *hnf1b* and *meis3* were largely unaffected (j). (k) Unilateral injection of *meis3*MO (40 ng) at the 2-cell stage caused a reduction of *egr4* and *mafb* expression*.* (l) Egr4 knockdown (*egr4*MO1; 40 ng) has no effect on *snail2* and *sox9* expression at the neural plate border. In all panels (d–l), the graphs indicate the percentage of embryos with normal (white), reduced/lost (red) or expanded/ectopic (blue) gene expression. The number of embryos analyzed is indicated on top of each bar. Dorsal views, anterior to top. (m) At stage 25 *egr4*MO1-injected embryos (40 ng) show a specific loss of anterior branchial neural crest stream that normally travels to the third branchial arch (red arrows). Lateral views, anterior to left (uninjected side; black arrows) or to right (injected side; red arrows), dorsal to top. Panels (d–m) scale bars, 300 μm. (n) Transverse section through an embryo injected with 40ng of *egr4*MO1. On the injected side (red arrow) the otic vesicle failed to form. The otic vesicle on the control side is indicated (black arrow). hb: hindbrain; ov: otic vesicle.

To determine whether Egr4 function is required for posterior hindbrain development we performed Egr4 knockdown in developing embryos using MOs. The first MO (*erg4*MO1) was designed to specifically interfere with translation of *egr4* mRNA. This was confirmed in an *in vitro* transcription/translation assay in which *erg4*MO1 blocked Egr4 protein production (Fig 3a; Supplementary Figure 1). Unilateral injection of *egr4*MO1 at the 2-cell stage led to a reduction of *krox20* expression in r5 at stage 15, without affecting *krox20* expression in r3; however, r3 appeared shifted posteriorly in some embryos (Fig. 3d). *egr4* knockdown also strongly inhibited the expression of *mafb* in r5/r6 (Fig. 3e). In morphant embryos the loss of *mafb* expression in r5/r6 was associated with a reduction of *hoxa3* expression, a direct target of Mafb^23^ (Fig 3f). The loss of *mafb* and *krox20* in morphant embryos could be rescued by injection of mRNAs encoding the hormone inducible version of *Xenopus egr4* (*egr4*GR) or mouse Egr4 (mEgr4GR) (Fig. 3e, g). To confirm the specificity of the knockdown phenotype, we used a second MO (*egr4*MO2) that specifically interfered with *egr4* pre-mRNA splicing by targeting the splice donor site of exon 1 (Fig 3b), resulting in the production of a transcript of higher size, due to intron retention (Fig 3c). The phenotype of *egr4*MO2-injected embryos was indistinguishable from the phenotype generated by injection of the translation blocking MO, with strong inhibition of *mafb* and *krox20* expression in r5/r6 and r5, respectively (Fig 3h). Furthermore, coinjection of the two MOs at low doses showed an additive effect on the repression of *mafb* expression (Supplementary Figure 2). The similarity of the two MO phenotypes, and the ability to rescue *krox20* and *mafb* expression in morphant embryos vouch for a specific requirement of Egr4 in posterior hindbrain development.

To evaluate the consequences of the loss of *krox20* and *mafb* on other regions of the hindbrain, we analyzed the expression of *hoxb1*, *fgf3* and *hnf1b* in morphant embryos. We found that the expression domain of *hoxb1* and *fgf3,* normally confined to r4, was expanded posteriorly (Fig 3i), consistent with a loss of r5 identity. The expression of *hnf1b* was largely unaffected, although slightly shifted posteriorly (Fig 3j), suggesting that Egr4 is functioning downstream of hnf1b. The transcription factor Meis3 is expressed in r2-r4^24^ and has been shown to be acting downstream of Pax3 and Zic1^25^. *meis3* expression level was unchanged in egr4-depleted embryos, however there was a posterior shift of its anterior boundary of expression, similar to the one observed for *hnf1b* (Fig 3j). We also analyzed the regulation of *egr4* expression by Meis3. Meis3 MO-mediated knockdown resulted in a severe reduction of *egr4* and *mafb* expression in r5/r6 (Fig 3k), suggesting that Meis3 is acting upstream of Egr4 and Mafb. This result is consistent with a previous study showing that Meis3 regulate cell fates in the hindbrain in a non-cell autonomous manner^24^.

### Egr4-depleted embryos have neural crest and inner ear defects

Because *egr4* expression at the neural plate border overlaps with the neural crest territory (Fig 2c), we analyzed the expression of two early neural crest transcriptional regulators, *snail2* and *sox9,* in morphant embryos. We found that both genes were largely unaffected in Egr4-depleted embryos (Fig 3l), suggesting that egr4 knockdown did not interfere with neural crest specification. In *Xenopus*, neural crest cells at hindbrain level migrate towards the branchial arches into four streams, the mandibular, hyoid, anterior and posterior branchial neural crest^26^. The stream of neural crest expressing *egr4*, completely overlaps with *krox20*-positive neural crest cells (Fig 2a), which corresponds to the anterior branchial stream that travels caudal to the otic vesicle (centered onto r4 in *Xenopus*^27^) into the third branchial arch^28^. In *egr4*-depleted embryos this stream of neural crest cells was specifically reduced or lost, as revealed by the expression of *krox20*, *sox10* and *tfap2α* at the tailbud stage (Fig 3m). Interestingly, even in morphant embryos retaining some *krox20* expression in r5, the stream of neural crest cells adjacent to this axial level was often missing, suggesting that both domains are regulated by different levels of *egr4*. The Mafb mouse mutant *Kreisler,* and its counterpart in fish *valentino*, which exhibit mis-patterning of hindbrain caudal to r3, have severe inner ear defects^23^,^29^,^30^. Since *egr4*-depleted embryos fail to express *mafb*, we analyzed otic vesicle formation in *egr4* morphant embryos by histology. We found that a majority of these embryos had reduced or missing otic vesicles on the injected side (Fig 3n). We posit that the neural crest and the inner ear phenotypes observed in *egr4*-depleted embryos could be the result of defects in posterior hindbrain patterning.

### Hnf1b activates *egr4* expression in r5/r6

**Figure 4:**
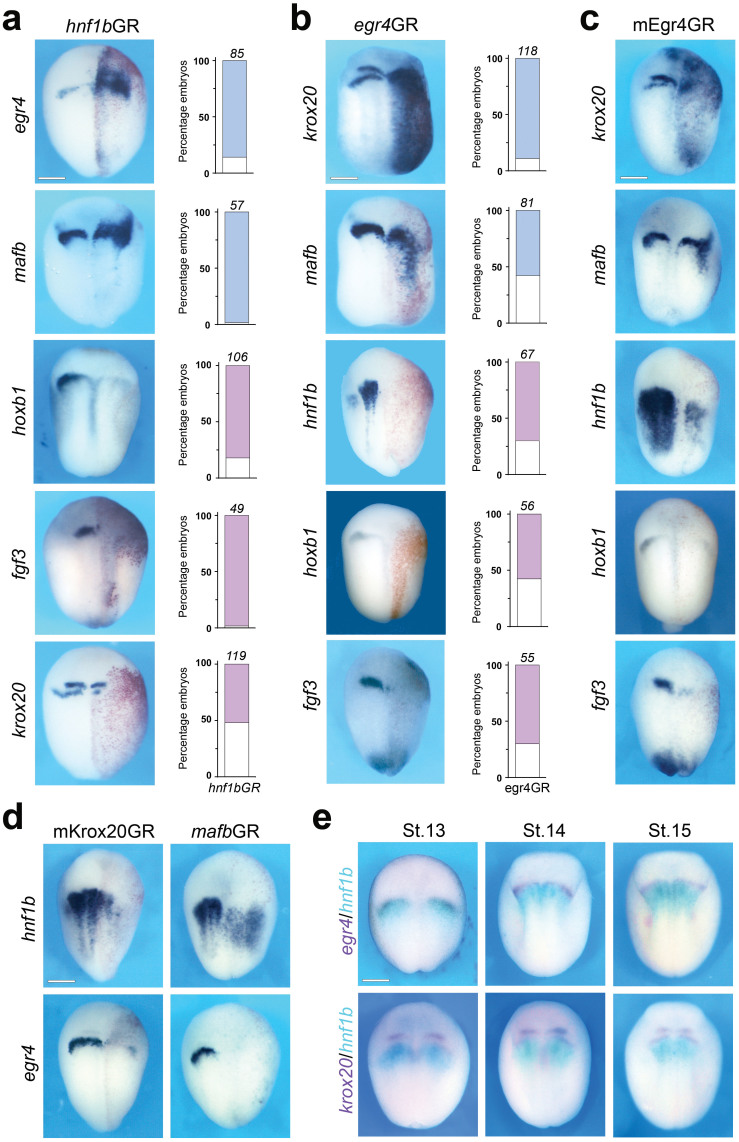
Cross-regulation of Hnf1b and Egr4. (a) Injection of 100 pg *hnf1b*GR mRNA expands *egr4* and *mafb* expression anteriorly, while repressing *hoxb1* and *fgf3* expression. (a) Injection of 100 pg *egr4*GR mRNA expands *krox20* and *mafb* expression and represses *hnf1b*, *hoxb1* and *fgf3* expression. The graphs indicate the percentage of embryos with normal (white), reduced/lost (red) or expanded/ectopic (blue) gene expression. The number of embryos analyzed is indicated on top of each bar. (c) Similar results were obtained by injection of 100 pg mouse Egr4 (mEgr4GR) mRNA. (d) Embryos injected with 100 pg of mouse Krox20 (mKrox20GR) or *Xenopus* *mafb* (*mafb*GR) mRNAs exhibit a dramatic reduction of *hnf1b* and *egr4* expression. (e) Two-color *in situ* hybriidization for *egr4*/*hnf1b* and *krox20*/*hnf1b*. Overtime, the anterior limit of *hnf1b* retracts posteriorly and segregates from the r5 *egr4* and *krox20* expression domains. Dorsal views, anterior to top. In all panels scale bars, 300 μm.

Hnf1b/vHnf1 is a major regulator of posterior hindbrain development where it participates in the formation of the boundary between r4 and r5 and establishes r5 and r6 identities. More specifically, Hnf1b represses r4 identity in r5/r6, and synergizes with r4-derived Fgf3 signal to drive *mafb* expression in r5/r6, and cooperate with Mafb for the direct transcriptional activation of *krox20* in r5^10^,^11^. To determine the position of Egr4 in this regulatory cascade we performed gain-of-function experiments by injection of mRNAs encoding a hormone inducible version of *hnf1b* (*hnf1b*GR). Unilateral injection of *hnf1b*GR mRNAs at the 2-cell stage caused an anterior expansion of *egr4* and *mafb* expression domains*,* associated with a down-regulation of *hoxb1* and *fgf3* (Fig 4a), indicative of a loss of r4 identity. The expression of *krox20* was reduced in these embryos (Fig 4a), since r4-derived Fgf signals are required to maintain *krox20* expression in r5^31^,^32^. These results suggest that *egr4* is activated by Hnf1b to establish r5 identity. Consistent with this view, *egr4* gain-of-function by injection of *Xenopus* *egr4* (*egr4*GR) or mouse Egr4 (mEgr4GR) mRNAs caused a dramatic expansion of *krox20* and *mafb* expression domains, often extending to the entire injected side (Fig 4b–c). The expression of *hnf1b*, *hoxb1*, and *fgf3* was strongly repressed in these embryos (Fig 4b–c).

The repression of *hnf1b* in Egr4GR-injected embryos suggested the existence of a negative feedback loop in the regulation of *hnf1b* expression in r5/r6 that may involve Egr4 directly or other factors activated by Egr4. To test this possibility, embryos were injected with mRNAs encoding a hormone inducible version of mouse Krox20 (mKrox20GR) or *Xenopus* *mafb* (*mafb*GR), and in both cases we observed a strong inhibition of *hnfb1* expression as well as a loss of *egr4* on the injected side (Fig 4d), indicating that Krox20 and Mafb are the likely factors repressing *hnf1b* expression in r5/r6. In most vertebrates, Hnf1b is initially expressed in the posterior hindbrain with an anterior limit at the r4/r5 boundary^33^,^34^,^35^. Later, as Krox20 is activated in r5 this anterior limit progressively retracts posteriorly, and *hnf1b* is eventually excluded from r5 and r6^21^,^33^,^34^,^35^. We performed two-color *in situ* hybridization to assess the spatio-temporal expression of *egr4* and *hnf1b*. At stage 13, *egr4* overlaps with *hnf1b* expression domain anteriorly, and as development proceeds, the anterior limit of *hnf1b* is shifted posteriorly and segregates from *egr4* expression domain (Fig 4e).

### Egr4 directly regulates *mafb* expression

**Figure 5:**
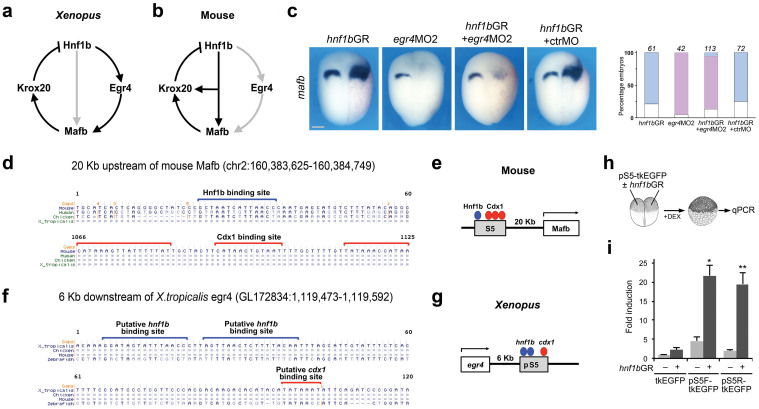
Hnf1b directly activates *egr4* expression. (a) In *Xenopus* Hnf1b induces *mafb* and *krox20* indirectly through the activation of *egr4*. (b) In the mouse Hnf1b directly activates *mafb* and *krox20* expression. (c) The expansion of *mafb* in embryos injected with *hnf1b*GR mRNA (10 pg) is completely blocked by co-injection of *egr4*MO2, but not by a control morpholino (ctrMO). Scale bar, 300 μm. The graph indicates the percentage of embryos with normal (white), reduced/lost (red) or expanded (blue) *mafb* expression. The number of embryos analyzed is indicated on top of each bar. (d) A conserved region upstream of the Mafb gene containing and Hnf1 binding site has been characterized as an r5/r6-specific enhancer (S5). (f) A putative S5 enhancer (pS5) downstream of *Xenopus tropicalis* *egr4* containing two putative Hnf1 binding sites was identified using rVISTA 2.0. (e, g) Schematic representation of the S5 and pS5 enhancer around *Mafb* (mouse) and *egr4* (*Xenopus*) genes, respectively. (h) Experimental procedure to test the activity of the pS5 in *Xenopus* embryo. (i) Fold EGFP induction analyzed by qPCR. Values are normalized to *ef1α*. Graph represents mean ± S.E. of 3 independent experiments. *, *P*<0.05; **, *P*<0.01; versus embryos injected with each vector alone. Student's *t*-test.

Our results suggest that Hnf1b induces *mafb* and *krox20* expression indirectly through the activation of Egr4 (Fig 5a). This is in contrast to what has been described in the mouse in which Hnf1b has been shown to directly activate *Mafb* expression^34^,^36^ (Fig 5b). To determine whether the activation of *mafb* by Hnf1b is primarily mediated through Egr4 in *Xenopus,* we evaluated the ability of *hnf1b*GR to activate *mafb* in the absence of Egr4 function. Co-injection of *egr4*MO2 with *hnf1b*GR mRNA completely blocked endogenous as well as ectopic *mafb* expression by Hnf1b (Fig. 5c). These results support the view that Hnf1b activates *mafb* via Egr4 in *Xenopus*.

In the mouse, Hnf1b directly binds with an evolutionally conserved region of DNA upstream of the *Mafb* gene, known as the S5 enhancer^34^,^36^. This region also contains three Cdx1 binding sites. In the hindbrain Cdx1 has an anterior limit of expression at the prospective r6/r7 boundary, and Cdx1 represses *Mafb* expression in this region, leading to the characteristic r5/r6 expression of *Mafb*^36^. The S5 enhancer has been mapped to a region 20 Kb upstream of the mouse *Mafb* gene. Sequence alignments indicate that this regulatory module is well conserved in human and chicken. A similar region could not be found around *Xenopus tropicalis* mafb gene using the UCSC Gene browser (Fig 5d,e). However, a region with similar characteristics, containing two putative Hnf1b binding sites and a single putative Cdx1 binding site, was identified 6Kb downstream of *Xenopus tropicalis* *egr4* gene (Fig 5f,g), here referred as a putative S5 enhancer (pS5). To determine whether this regulatory module can drive *egr4* expression, pS5 was cloned in forward (pS5F) and reverse (pS5R) orientation into ptkEGFP vector containing a basal thymidine kinase promoter driving enhanced GFP expression^37^. Both constructs, pS5F-ptkEGFP and pS5R-ptkEGFP, were co-injected with *hnf1b*GR mRNA at the 2-cell stage and immediately treated with dexamethasone. Embryos were cultured for 6 hours and analyzed by qPCR (Fig 5h). We observed a significant up-regulation of EGFP transcripts in embryos co-injected with *hnf1b*GR mRNA and pS5F-ptkEGFP (4.6 fold) or pS5R-ptkEGFP (10.2 fold) as compared to embryos injected with each vector alone (Fig 5i). These results indicate that Hnf1b may directly activate *egr4* expression via the pS5 enhancer.

## Discussion

**Figure 6:**
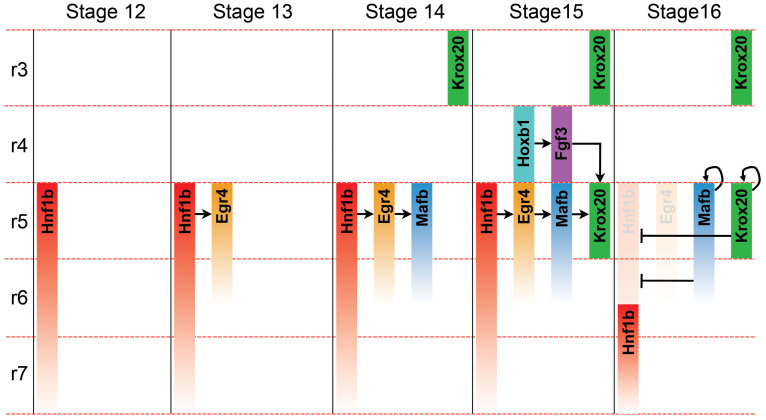
Model for the temporal regulation of r5 identity by Hnf1b, Egr4, Mafb and Krox20 in *Xenopus* posterior hindbrain.

Our findings reveal a novel function for Egr4 in posterior hindbrain development. Egr4 is specifically and transiently activated in r5/r6 by Hnf1b, and is required for *mafb* and *krox20* expression in r5/r6 and r5, respectively (Fig 6). In addition, Egr4 knockdown leads to a specific loss of the anterior branchial neural crest stream that normally travels to the third branchial arch, and inner ear defects. While a recent study has implicated Egr4 in the differentiation of the brain primordia during planarian regeneration^38^, our study is the first report describing a role for Egr4 in the vertebrate nervous system.

Using a combination of gain- and loss-of-function approaches we provide evidence that Egr4 is part of a conserved regulatory cascade, involving Hnf1b, Mafb and Krox20, essential to specify r5 identity in the hindbrain. Hnf1b/vHnf1 is a major regulator of posterior hindbrain development. It is believed to repress r4 identity in r5/r6, and to synergize with r4-derived FGF signals to directly drive *mafb* expression in r5/r6. Hnf1b eventually cooperates with Mafb for the direct transcriptional activation of *krox20* in r5^10^,^11^. Our gain-of-function studies indicate that Hnf1b can promote *egr4* and *mafb* expression. Moreover, ectopic expression of *egr4* is sufficient to activate *mafb* and *krox20* in the embryo, suggesting that Hnf1b may control the expression of these factors indirectly in *Xenopus*, through the activation of Egr4. Consistent with this possibility, *hnf1b*GR is unable to rescue *mafb* expression in Egr4-depleted embryos. This is in contrast to what has been reported in other species in which Hnf1b directly activates *Mafb* and *Krox20* expression^11^,^21^,^36^,^39^. Once activated by Hnf1b in r5/r6, Egr4 induces the expression of *mafb1* and *krox20*, which in turns act as negative regulators of Hnf1b to restrict its expression to a level posterior to r6. Accordingly, ectopic expression of Krox20 or Mafb represses *hnf1b* expression in the caudal hindbrain. The inhibitory activity of Krox20 in restricting Hnf1b expression posteriorly has been well described in other species^33^,^34^,^35^. This negative feedback loop may also account for the transient expression of *egr4* in r5/r6 in *Xenopus*, which appears to require continued Hnf1b input for its maintenance. The expression of Krox20 and Mafb1 is maintained primarily through an autoregulatory mechanism^36^,^39^,^40^ (Fig 6).

Egr4 belongs to the early growth response factor family of transcription factors. Members of this family were initially identified as genes rapidly induced by growth factors. A defining feature of Egr proteins is a highly conserved DNA-binding domain composed of three zinc finger motifs that bind a 9-bp response element to initiate transcriptional response^41^. Egr2/Krox20, the most extensively studied member of this family, is essential for the development of r3/r5 by directly activating Hoxa2 and Hoxb2 in r3/r5 and Hoxb3 in r5^42^,^43^,^44^. Egr2/Krox20 mutant mouse embryos also have peripheral nerve myelination defects^45^. Egr1/Krox24 plays a critical role in regulating the luteinizing hormone (LH) β gene expression; Egr1-deficient mice have low levels of LHβ in the pituitary and low levels of LH in serum, and this appears to primarily affect female fertility^46^,^47^. Egr3 is required for muscle spindle morphogenesis and normal proprioception in mice^48^.

Egr4 is detected at low levels in testicular germ cells and Egr4-deficient mice are characterized by a defect in germ cell maturation, leading to male infertility^49^. Egr4 is also expressed at high level in postnatal rat brain^50^, and at E13.5 in the mouse spinal cord^51^. Beside this report, there is no detailed description of Egr4 developmental expression in the mouse. Egr4 mutant mouse embryos exhibit no obvious phenotypic abnormalities in the nervous system. However, embryos have not been examined specifically for defects in the developing brain. It is possible that the effects may be transient or subtle. Alternatively, Egr4 may not be an essential component of the regulatory cascade controlling r5 identity in mammals. Interestingly, mouse Egr4 can fully compensate for the loss of Egr4 in frog embryos, and in overexpression studies its function is indistinguishable from that of *Xenopus* Egr4. Therefore, the difference in the regulation of r5 identity between frog and mouse cannot be explained by intrinsic functional differences in the Egr4 proteins.

In the mouse, Hnf1b regulates Mafb expression through the S5 enhancer^36^, a region highly conserved in chicken and human. This enhancer could not be identified around *Xenopus tropicalis mafb* gene. However, we found that a regulatory module similar to the S5 enhancer, containing two Hnf1b binding sites, was present downstream of the *egr4* gene in *Xenopus tropicalis*, a region also conserved in zebrafish. Using a reporter assay in the embryo we were able to show that Hnf1b can activate this putative enhancer. We propose that rearrangements in the regulatory regions around *egr4* and *mafb* genes may account for the evolutionary changes in the importance of Egr4 in establishing r5 identity in the hindbrain of amniotes versus anamniotes.

## Experimental Procedures

### Plasmid constructs and morpholino antisense oligonucleotides

The full-length *Xenopus laevis egr4* was obtained by RACE-PCR using SMARTer^TM^ RACE cDNA amplification Kit (Clontech, Mountain View CA) according to the manufacturer's instructions. Briefly, a first-strand cDNA was synthesized from 1 μg of total RNA isolated from neurula stage embryos. Two sets of gene-specific primers (GSP), 5′-RACE-*egr4* and 3′-RACE-*egr4* (5′-AGCAAGGGCTGTGGCGACTTGACTGT CT-3′ and 5′-AGCCGAAGCGTCAAAGTGGGAAACGCGA-3′) were designed based on EST sequences (Unigene ID Xl.53808). The RACE-PCR conditions were as follows: 5 cycles at 94°C for 30 sec and at 72°C for 3 min; 5 cycles at 94°C for 30 sec, at 70°C for 30 sec, and 72°C for 3 min; 27 cycles at 94°C for 30 sec, at 68°C for 30 sec, and 72°C for 3 min. 5′-RACE and 3′-RACE PCR products were cloned into pGEM-T Easy vector (Promega, Madison WI) and sequenced. The sequence of *Xenopus egr4* has been deposited into GeneBank (Accession # KF957598). The ORF of *Xenopus laevis* *hnf1b* and *mafb* were amplified by PCR from neurula stage embryo cDNA using primers based on published sequences (GeneBank accession # NM_001089811 and NM_001090383). Mouse Egr4 was purchased from Open Biosystems and mouse Krox20 was a gift of Drs. Schneider-Maunoury and Gilardi-Hebenstreit^52^. Hormone-inducible constructs were generated by sub-cloning the coding region of each gene into pCS2+GR^53^. These constructs are referred as *egr4*GR, *hnf1b*GR, *mafb*GR, and mKrox20GR. Pax3 (*pax3*MO^15^;), Zic1 (*zic1*MO^16^;), Meis3 (*meis3*MO^24^;) and Egr4 (*egr4*MO1; CCTGGCAGGAGAGGTCCATCGT TAT, *egr4*MO2; GTCCTTACCTG ACCTGAGCTGAGT) MOs were purchased from GeneTools. The specificity of the *egr4*MO1 was tested in an *in vitro* transcription/translation coupled rabbit reticulocyte lysate assay (Promega, Madison WI) as previously described^54^. Synthetic mRNAs encoding *pax3*GR, *zic1*GR, *egr4*GR, *hnf1b*GR, *mafb*GR, mEgr4GR, mKrox20GR and β-galactosidase were synthesized *in vitro* using the Message Machine kit (Ambion-Life Technologies, Grand Island NY).

### Embryos, injections and explants culture

*Xenopus laevis* embryos were staged as previously described^55^ and raised in 0.1X NAM (Normal Amphibian Medium^56^;). This study was performed in accordance with the recommendations of the Guide for the Care and Use of Laboratory Animals of the National Institutes of Health. The animal protocol (# 120311) was approved by the Institutional Animal Care and Use Committee of New York University. Embryos were injected in one blastomere at the 2-cell stage and analyzed by *in situ* hybridization at stage 15. MOs (40–50 ng) and synthetic mRNA (100 pg) were injected together with 500 pg of β-galactosidase mRNA as a lineage tracer. Embryos injected with mRNA encoding the hormone inducible version of *Xenopus* *egr4* (*egr4*GR), *hnf1b* (*hnf1b*GR) and *mafb* (*mafb*GR), and mouse Egr4 (mEgr4GR) and Krox20 (mKrox20) embryos were treated at stage 12.5 with 10 μM dexamethasone (DEX; Sigma-Aldrich, St Louis MO) and collected at stage 15/17. For animal cap explant experiments, both blastomeres at the 2-cell stage were injected in the animal pole region 100 pg of mRNAs encoding *pax3*GR and *zic1*GR, explants were dissected at the late blastula stage and immediately cultured for several hours in NAM 0.5X plus 10 μM of dexamethasone. In some experiments, 10 μg/ml cycloheximide (CHX; Sigma-Aldrich, St Louis MO) was added to the culture medium to block protein synthesis, in which case explants were pretreated with cycloheximide for 30 min before dexamethasone treatment^53^. The explants were subsequently analyzed by quantitative PCR (qPCR).

### Lineage tracing, *in situ* hybridization and histology

*Xenopus* embryos were fixed in MEMFA and processed for Red-Gal (Research Organics, Clevelan OH) staining to visualize the lineage tracer (β-galactosidase) prior to *in situ* hybridization. Whole-mount *in situ* hybridization was performed as previously described^57^. Digoxygenin (DIG)- and fluorescein isothiocyanate (FITC)-labeled antisense RNA probes (Roche Diagnostics, Indianapolis, IN) were synthesized using template cDNA encoding Zic1^14^, Pax3^14^, En2^17^, Krox20^28^, Mafb^58^, Hnf1b^59^, Meis3^24^, Fgf3^60^, Hoxb1^61^, Hoxa3^62^, Snail2^22^, Sox9^63^, Sox10^64^ and Tfap2a^65^. For double in situ hybridization DIG- and FITC-labeled probes were hybridized simultaneously and sequentially detected using anti-FITC and anti-DIG alkaline phosphatase conjugated antibodies. FITC-labeled probe was visualized first using 4-toluidine salt (BCIP; Roche Diagnostics, Indianapolis, IN) and after inactivation of the anti-FITC antibody, the color reaction for the DIG-labeled probe was performed using Magenta Phosphate (5-bromo-6chloro-3indoxyl phosphate; Biosynth, Itasca IL). For histology, fixed embryos were embedded into Paraplast+, 12 µm sections cut on a rotary microtome (Olympus, Center Valley PA) and counterstained with Hematoxylin.

### RNA preparation and qPCR

Total RNA from whole embryos was extracted using RNeasy Micro Kit (Qiagen, Valencia CA) according to the manufacturer's instructions. During the extraction procedure, the samples were treated with DNase I to eliminate possible genomic DNA contamination. The amount of isolated RNA was quantified using a Nanodrop spectrophotometer (Nanodrop Technologies; Wilmington, DE). For mRNA extraction from animal cap explants, pools of 8 explants were homogenized and mRNA was isolated using Dynabeads® mRNA DIRECT^TM^ Micro Kit (Invitrogen, Grand Island NY). cDNA synthesis from total RNA (whole embryos) and mRNA (animal cap explants) were performed using Superscript VILO cDNA Synthesis Kit (Invitrogen, Grand Island, NY) according to the manufacturer's instructions. Quantitative PCR was performed on an Eco Real-Time PCR System (Illumina, San Diego CA) using the primers shown in Table 1 and the *Power* SYBR Green PCR Master Mix (Invitrogen, Grand Island NY). The reaction mixture consisted of 10 µl of *Power* SYBR Green PCR Master Mix, 200 nM primers, and 2 µl of cDNA in a total volume of 10 µl. The PCR conditions were as follows: incubation at 50°C for 2 min; activation at 95°C for 10 min; 40 cycles at 95°C for 10 s and at 60°C for 30 s; melt curve at 95°C for 15 s, 55°C for 15 s, and at 95°C for 15 s. The ΔΔCT method was used to quantify the qPCR results. Each reaction included a standard curve of serial dilution points (in 10-fold increments) of test cDNA. In each case, *ornithine decarboxylase 1* (*odc1*) or *elongation factor 1α* (*ef1α*) was used for normalization.

### Isolation of conserved regions

Using the UCSC Gene browser, we identified regions in *Xenopus tropicalis* genome containing two putative Hnf1b binding sites and a cdx1 binding site in the vicinity of Egr4 coding region (pS5). Using PCR, we amplified this putative regulatory region from *Xenopus tropicalis* genomic DNA. The pS5 module was cloned in forward and reverse orientation upstream of ptkEGFP vector that contains the Herpes simplex virus thymidine kinase basal promoter driving enhanced GFP expression^37^. These constructs are referred as pS5F-ptkEGFP and pS5R-ptkEGFP, respectively.

## Author Contributions

C.-J.B. and J.-P.S.-J. designed the experiments, prepared the figures and wrote the manuscript. C.-J.B., J.J. and J.-P.S-J. performed the experiments and analyzed the data.

## Supplementary Material

Supplementary Information Supplementary Figures

## Figures and Tables

**Table 1:** Primers for qPCR

Gene	Forward primer	Reverse primer
*hnf1b*	5′-GTTCTGTTCCCAGTTGTTTGTT-3′	5′-GGATATACCACGTGAGCTTCTG-3′
*egr4*	5′-AACGCGAGACCGTGTTATT-3′	5′-GGAACAGAAAGTGCGTGTAAATAG-3′
*krox20*	5′-TCTCCTACTCGTCCAACTACC-3′	5′-CGCTCACTAGATTGAAGATCCC-3′
*mafb*	5′-GACGCAGTAGAAGCCCTTATT-3′	5′-GATGCTGGTTCTGGTGATGA-3′
*odc1*	5′-ACATGGCATTCTCCCTGAAG-3′	5′-TGGTCCCAAGGCTAAAGTTG-3′
*ef1*α	5′-ACCCTCCTCTTGGTCGTTTT-3′	5′-TTTGGTTTTCGCTGCTTTCT-3′
*egfp*	5′-GAACCGCATCGAGCTGAA-3′	5′-TGCTTGTCGGCCATGATATAG-3′

## Notes

**Funding** Work was funded by a National Institutes of Health (NIDCR) grant [R01-DE014212] to J-P. S-J.
