# Supplementary material for: A novel function for Egr4 in posterior hindbrain development
Source: Sci Rep. 2015 Jan 13;5:7750. doi: 10.1038/srep07750 (PMC4291570; doi:10.1038/srep07750)

Ms: SREP-14-08303-T

Supplementary Information

A novel function for Egr4 in posterior hindbrain development

Chang-Joon Bae, Juhee Jeong and Jean-Pierre Saint-Jeannet

Supplementary Figure 1:

Increasing amounts of *egr4*MO1, 10 ng (+), 100 ng (++), and 1000 ng (+++) blocks translation directed by *egr4* mRNA in an *in vitro* coupled transcription/translation reaction. Full blott of the cropped version presented in Figure 3a.

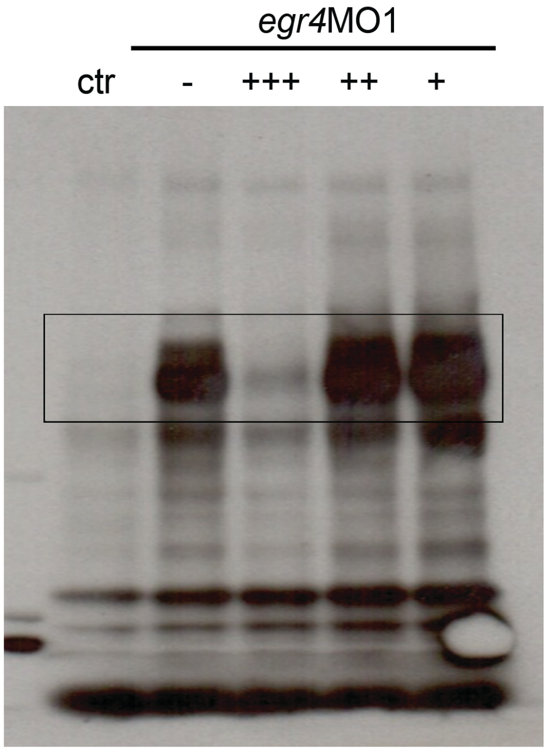

Supplementary Figure 2:

The coinjection of low doses of translation (egr4MO1) and splice (egr4MO2) blocking antisense shows an additive effect on the repression of *mafb* expression at the neurula stage. The graph indicates the percentage of embryos with normal (white), reduced/lost (red) or expanded/ectopic (blue) gene expression. The number of embryos analyzed is indicated on top of each bar.

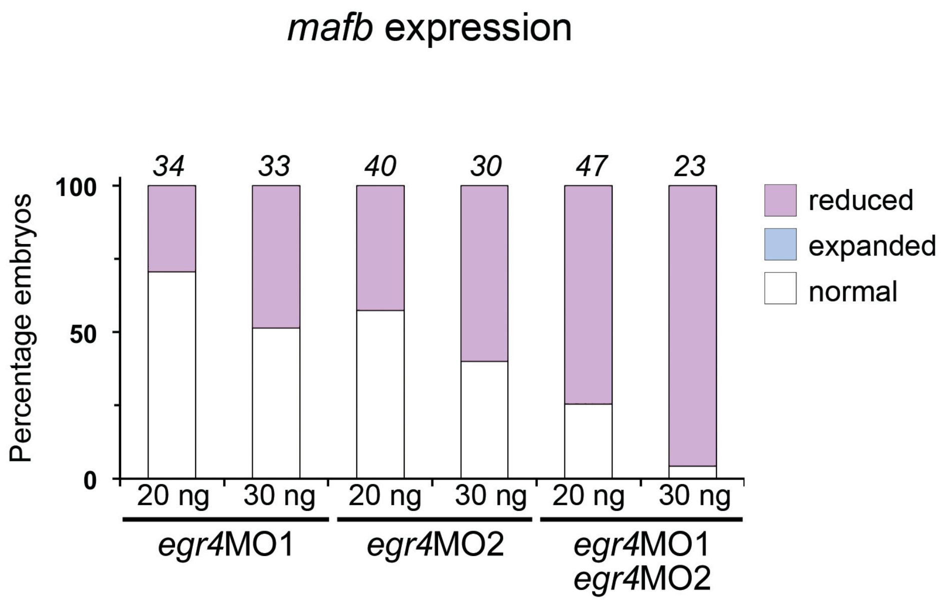

Supplement: Supplementary Information — Supplementary Figures [file srep07750-s1.pdf]
